# Early colonic-preparation and salvage laparoscopic appendectomy (ECSLA)- innovative protocol for the management of magnets ingestion

**DOI:** 10.1186/s12245-024-00678-2

**Published:** 2024-07-15

**Authors:** Ortal Schaffer, Adi Kenoshi, Osnat Zmora

**Affiliations:** 1 Shamir medical center, Department of Pediatric Surgery, Zerifin, Israel; 2 Faculty of Medicine, Tel Aviv University, Tel Aviv, Israel

**Keywords:** Magnets, Ingestion, Pediatric

## Abstract

**Background:**

Ingestion of magnets carries risks for significant morbidity. We propose a new protocol designed to reduce the need for surgery, shorten length of stay, and decrease morbidity.

**Methods:**

The Early Colonic-preparation and Salvage Laparoscopic Appendectomy (ECSLA) protocol includes initiating colonoscopy preparation upon admission in asymptomatic patients if magnets are not amenable to removal by gastroscopy, and laparoscopic magnets retrieval via appendectomy if surgery is eventually needed. The protocol was initiated in May 2023. A retrospective study of all cases of ingested magnets in children in our institution during July 2020 – January 2024 was conducted to retrieve and analyze demographic, clinical, imaging, management, and outcome data.

**Results:**

During the 3.5-year study period, 13 cases of ingested multiple magnets were treated, including 7 cases since initiation of ECLSA protocol, with no complications. Since initiation of ECSLA protocol, Early colonic preparation resulted in spontaneous passage of magnets (two cases) and successful colonocsopic removal (three cases), with two cases in which magnets were retrieved via gastroscopy upon admission, and no patients needing surgical intervention. Length of stay (LOS) was short (1–3 days).

**Conclusions:**

The ECSLA protocol is a promising tool for preventing surgical intervention and complications and for possibly shortening LOS in children who have ingested multiple magnets.

## Background

Foreign body ingestions are frequent in childhood with most ingested foreign bodies passing spontaneously without complications. Since the first report of magnet ingestion by McCormick et al. (2002) [1], the incidence of ingestion of magnets has been rapidly increasing [2, 3]. Ingestion of earth magnets increases the risk of morbidity and mortality, with 57% of cases involving multiple magnets [1, 4]. The force of these magnets is frightful. After swallowing more than one magnet or a magnet with another metal object, the objects may exert strong attractive forces on each other despite being in different areas of the bowel [5–7]. In this scenario, two segments of bowel can adhere to each other with significant strength, which may result in ischemia, pressure injuries, bowel perforation, fistula formation, and volvulus. This may lead to serious outcomes, including sepsis and emergent intestinal resection [3, 8–11]. We know from previous studies that even if magnets are quickly removed from the lumen by endoscopy, indentations and ulceration of the mucosa may occur, sometimes in less than 8 h [12]. Most children are asymptomatic in the early phase of ingestion. However, complications can occur even when the child is asymptomatic. Therefore, the current protocols which usually recommend intervention only after symptoms have occurred or when a significant time since ingestion has passed [13, 14], may result in preventable morbidity.

In this manuscript, we aim to describe the evolution, formation, application, and outcomes of an innovative proactive protocol designed to prevent complications associated with ingestion of multiple magnets.

## Methods

We performed a retrospective study of all pediatric cases of ingested multiple magnets admitted to our institution during July 2020 - January 2024, before and after initiation of the Early Colonic-preparation and Salvage Laparoscopic Appendectomy (ECSLA) protocol in May 2023.

### ECSLA Protocol (Fig. 1)

**Fig. 1:**
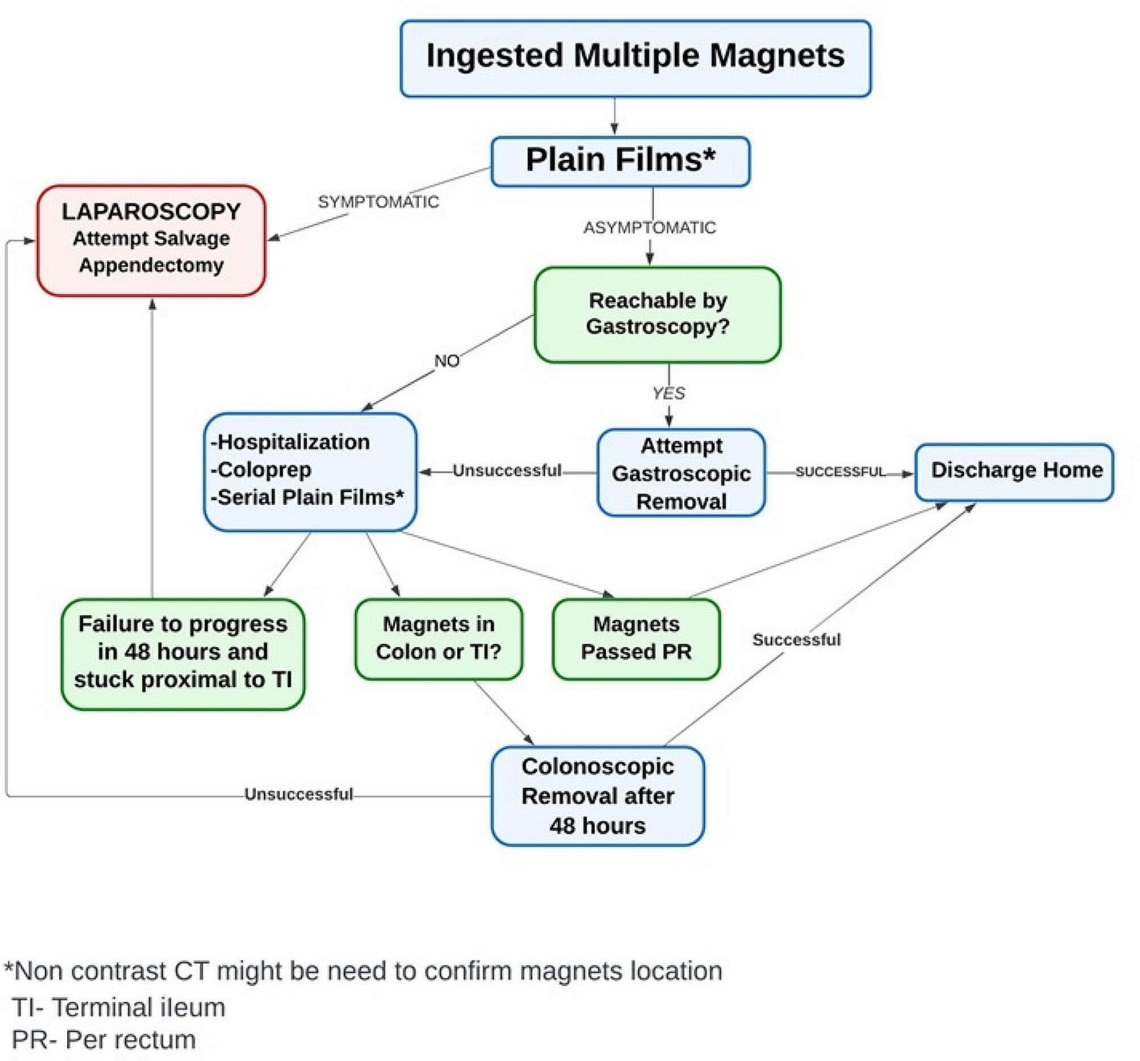
ECSLA protocol for management of multiple magnets ingestion

After initial assessment for number of ingested magnets, timing of ingestion and symptoms, a plain abdominal film is obtained. If magnets appear reachable by gastroscopy, gastroscopy is performed. Otherwise, or if gastroscopy fails, the patient is hospitalized. If the patient is symptomatic at any time, laparoscopy is performed. If the patient is asymptomatic, colonic preparation (Coloprep) for colonoscopy is started immediately upon admission. This may allow defecation of the magnets or make them reachable in another 48 h by colonoscopy. During hospitalization and preparation for colonoscopy, serial x-rays are taken every 8–12 h with three possible scenarios.

1. If the patient has defecated and magnets are no longer visible on X rays the child is discharged home.
2. If after 48 h the magnets fail to progress and are suspected to be halted in the terminal ileum/cecum, colonoscopy is performed for magnets removal.
3. If after 48 h the magnets fail to progress and are halted in a position proximal to the terminal ileum laparoscopy is performed. The laparoscopoic technique involves using the magnetic forces between the magnets and the laparoscopic instruments to mobilize the magnets into the appendix and accomplish an appendectomy with evacuation of magnets through the appendix. If laparoscopic mobilization of magnets fails, laparoscopic assisted mobilization and appendectomy are attempted with enlargement of the umbilical incision, partial bowel evisceration and manual mobilization of magnets into the appendix. Intra-operative findings might exclude salvage appendectomy and necessitate other surgical steps.

Of note, if at any time pre-intervention magnet localization is unclear using plain abdominal films, a non- contrast CT might be indicated to better determine the location of magnets and thus assist with decision regarding the appropriate method of intervention.

Demographic, clinical, imaging, management, and outcome data were collected and analyzed. Outcome data included mode of magnets evacuation, length of stay (LOS) and complications.

The retrospective study was approved by the institutional review board in accordance with the Declaration of Helsinki as revised in 2013, approval #0226-23-ASF.

## Results

Over the 3.5-year study period 13 cases of ingested multiple earth magnets were treated in our institution. The demographic, clinical, imaging, management, and outcome data for the entire group are summarized in Table 1. Except for case #1, which was symptomatic though no history of foreign body ingestion was given, all other 12 cases were asymptomatic.

**Table 1:** Demographic, clinical, imaging, management, and outcome data for 13 cases

case#	age	sex	medical background	time from ingestion	symptoms	suspected initial location	imaging	intervention	final findings	LOS (d)	complications
1	10y	f	none	unknown	Consistent with suspected appendicitis	unknown	US, intraoperative fluoroscopy	lap converted to open appendectomy, suturing of deserozations	magnets in distal ileum and cecum, serosal tears	5	none
2	1y 9 m	m	none	3 weeks	Incidental findings in CXR (performed d/t fever and cough)	stomach	Abdominal film, CT	Failed gastroscopy; Lap converted to laparotomy, repair of perforations	magnetic gastro-jejunal fistula	8	none
3	8y	f	none	1.5 h	asymptomatic	stomach	abdominal film	failed gastroscopy; lap assisted jejunal enterotomy	magnets stuck in jejunum	7	none
4	9y	f	none	4 h	asymptomatic	stomach	abdominal films	Failed gastroscopy; Coloprep and colonoscopic evacuation	magnets in sigmoid	3	none
5	11y	f	none	1.5 h	asymptomatic	stomach	abdominal film	gastroscopy	magnets in stomach	1	none
6	16y	f	psychiatric	unknown	asymptomatic	small bowel	abdominal films, CT	lap assisted appendectomy	magnets in cecum	3	none
initiation of full ECSLA protocol											
7	16y	f	psychiatric, rec ingestion	unknown	asymptomatic	stomach	abdominal films, CT	failed gastroscopy, ECSLA, colonoscopic evacuation	magnets in cecum	2	none
8	15y	f	none	1.5 h	asymptomatic	small bowel	abdominal films	ECSLA, magnets passed PR	magnets passed PR	1	none
9	9y	m	none	2 h	asymptomatic	duodenum	abdominal films, CT	failed gastroscopy; ECSLA, colonoscopic evacuation	magnets in cecum	2	none
10	9y	f	none	3 h	asymptomatic	stomach	abdominal film	gastroscopy	magnets in stomach	1	none
11	9y	m	none	1 h	asymptomatic	stomach	abdominal film	gastroscopy	Magnets in duodenum	1	none
12	6y	m	none	1 h	asymptomatic	small bowel	abdominal film	ECSLA, magnets passed PR	magnets passed PR	2	none
13	14y	m	none	1 h	asymptomatic	small bowel	abdominal film, CT	ECSLA, colonoscopic evacuation	magnets in cecum	3	none

LOS- length of stay; d- days; y- years; f- female; US- ultrasound; m- months/male; CXR- chest X ray; d/t- due to; CT- computorized tomorgraphy; PR- per rectum

The first 6 cases were managed according to standard protocols [13, 14]- gastroscopic removal if magnets were observed in the stomach, observation with serial x rays for up to 48 h for asymptomatic patients, and surgical intervention if magnets have not progressed within 48 h. We have explored modifications to the standard protocols and added preparation for colonoscopy (Coloprep) in one case (case #4), starting on the second day of hospitalization, with resultant successful colonoscopic removal of magnets and discharge home after 3 days. Case #1 was significant for the surgical approach of using appendectomy for removing the magnets. In this case, the patient was taken to the operating room because of a working diagnosis of acute appendicitis, as no history of magnets ingestion was given. Since the appendix appeared normal at laparoscopy, laparoscopic inspection of the distal ileum was pursued, during which small bowel and the cecum were found to be magnetizing to each other and to the laparoscopic instruments. This prompted an intra-operative fluoroscopy, and the diagnosis of ingested magnets was made. Laparoscopic mobilization of the magnets into the appendix was not fully successful, and therefore the procedure was converted to an open procedure through a McBurney incision. Mobilization of the magnets into the appendix was completed manually and the magnets were removed via appendectomy. Additional suturing of cecal and distal ileal serosal tears was performed.

**Fig. 2:**
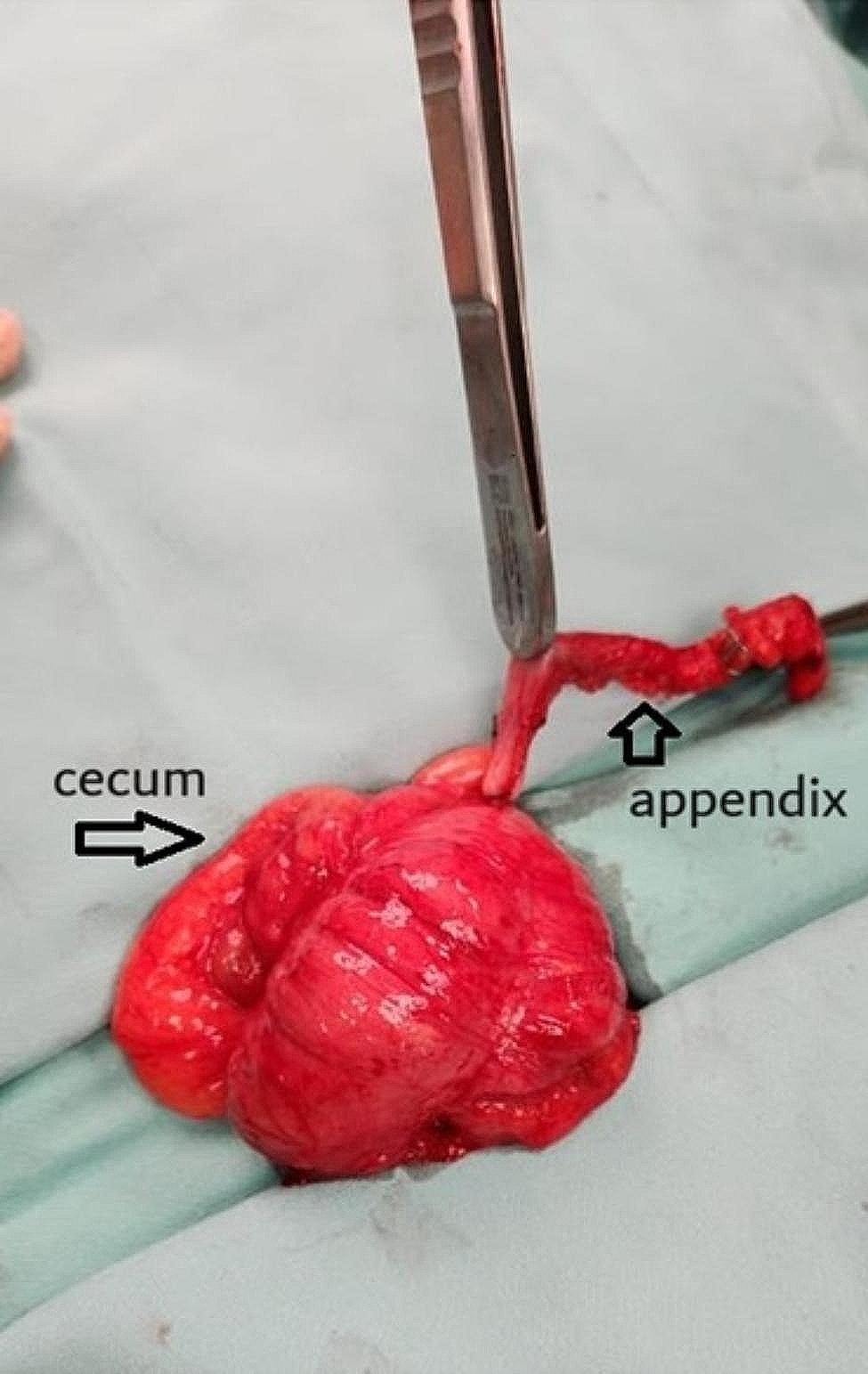
Magnets in the appendix attracted to a metal pickup (Case #6)

Case #6 led to the initiation of the full ECSLA protocol. In this case, gastroscopy was not pursued due to a more distal initial location of magnets. The magnets failed to progress within 48 h and were stuck in the terminal ileum/cecum. Since no colonic preparation was given, colonoscopy was not feasible. As the possibility of magnets being adherent to each other through bowel walls with possible bowel wall ischemia/necrosis was a viable option, surgical intervention was needed. During laparoscopy, salvage appendectomy was used for retrieval of magnets from the cecum. Due to technical difficulties of laparoscopic mobilization of magnets from the cecum to the appendix, the cecum was mobilized laparoscopically, the umbilical incision extended, the cecum exteriorized, and manual manipulation completed the mobilization of magnets into the appendix (Fig. 2). This case led to the initiation of the ECSLA protocol since a prepped colon could have enabled removal of the magnets via colonoscopy, thus obviating the need for surgery.

**Fig. 3:**
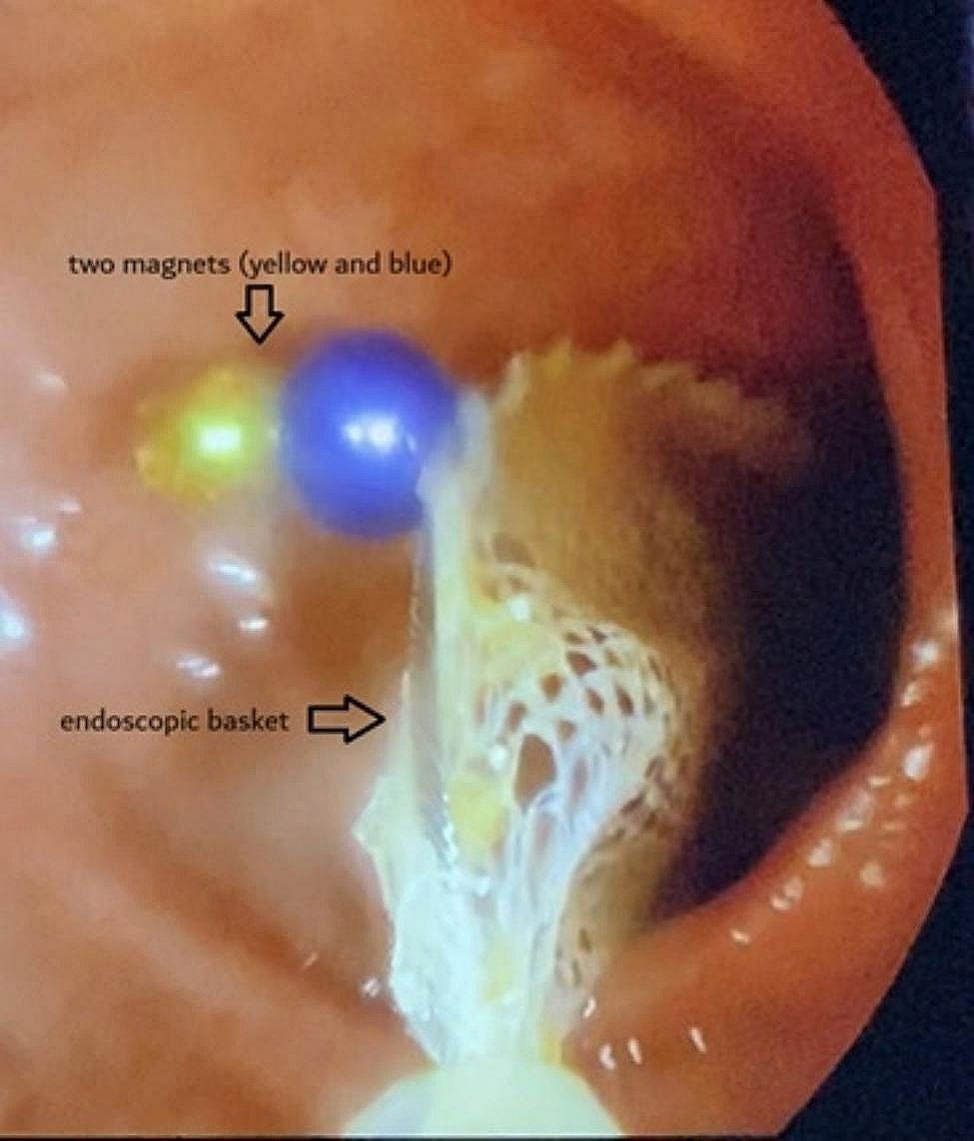
Two magnets attracted to an endoscopic basket during colonoscopic removal (case #9)

When the same patient presented two months later with another episode of ingesting multiple magnets (case #7) the ECSLA protocol was activated, enabling colonoscopic removal for no progression of magnets after 48 h, and preventing another surgery. Since the initiation of the ECSLA protocol, none of the seven patients underwent surgery. In two cases the magnets were passed spontaneously (cases #8, #12), in three cases the magnets were removed via colonoscopy [case#7, case #9 (Fig. 3), case #13], and in two cases magnets were removed by upper endoscopy upon admission.

No complications were noted in any of the cases included in the study. LOS was short when gastroscopy was successful in retrieval of magnets (cases #5, #10, #11, LOS of 1 day in each case). LOS with Early Coloprep alone was between 1 and 3 days (1–2 days with defecation of magnets following Coloprep, and 2–3 days with colonoscopic removal). When surgery was needed (all cases- prior to the initiation of the ECSLA protocol) LOS was longer (3, 5, 7, and 8 days).

## Discussion

In this report, we described the application of a new protocol for the management of ingested magnets. Application of Early Coloprep was associated with a short LOS with no reported complications and possibly obviated the need for surgery. Application of laparoscopic salvage appendectomy prevented bowel enterotomies and possibly large abdominal incisions with no reported complications.

The main advantage of Early Coloprep is the prevention of surgical intervention in an asymptomatic patient. When the location of magnets is amenable to endoscopic retrieval, NASPGHAN Endoscopy Committee recommends urgent endoscopic removal even for the asymptomatic child [15]. In practice, this recommendation applies only to upper endoscopy since colonoscopy is usually not successful, and not even attempted without appropriate colonic preparation.

The management of the asymptomatic patient with multiple magnets beyond the ligament of Treitz but proximal to the terminal ileum is guided by concerns for adherence of magnets to each other with an intervening bowel loop, which may lead to bowel necrosis, with subsequent bowel perforation or fistula formation [8–11]. Waters et al. conducted a survey of surgeons regarding magnet ingestions and recommended surgical management for all post pyloric magnets [16]. Hospitals equipped with small bowel enteroscopy capabilities can manage these cases with endoscopic removal [15] but most centers do not have this option available. Therefore, when the initial location of magnets is not amenable for retrieval via upper endoscopy, most protocols recommend serial x-ray follow-up and surgical intervention if there is failure of magnet progression after 48 h [13, 14].

In general, prokinetic agents and cathartics have not been shown to significantly improve gut transit time or facilitate the passage of foreign bodies [17]. However, some authors recommend PEG 3350 solution or other laxative preparations to aid in passage [6] and to help prepare for colonoscopy [12] in cases in which gastroscopic removal is not an option, however only after magnets have failed to progress on serial x-rays. The ECSLA protocol is unique by initiating Coloprep early upon admission, and therefore enabling colonoscopic removal or even spontaneous expulsion of magnets by the time point of 48 h.

Published protocols for management of ingested magnets do not specify the details of the surgical intervention when indicated. Our experience with case #1 who was taken to the operating room with the diagnosis of Acute Appendicitis with incidental findings of ingested magnets which were evacuated via the appendix initiated a literature review. The literature review resulted in a notion that salvage appendectomy might be an alternative way of magnets evacuation. There have been few case reports in the literature of laparoscopic appendectomy for magnets retrieval [18–20]. In most of these cases, the magnets were already lodged in the appendix, while in other cases the magnets were mobilized laparoscopically into the appendix. We suggest that using the appendix for evacuation of magnets in selected cases might obviate the need for enterotomies, thus decreasing the risk of enteric leaks [21], as it might obviate the need and for a possible larger abdominal incision. We further suggest that when laparoscopic mobilization is unsuccessful, lap assisted techniques through a small open incision and using manual magnet mobilization should be considered. Nevertheless, the disadvantages of amputating a healthy appendix as opposed to performing an enterotomy without bowel resection should be weighed against salvage appendectomy. Appendectomy should be avoided in children with anorectal malformations or other medical conditions that are associated with fecal or urinary incontinence, as the appendix may be needed in such children as a urinary conduit or as a conduit for antegrade enemas [22, 23]. In addition, a number of recent studies suggest that the appendix has several significant physiological roles, such as production of mesenchymal stem cells [24] and functioning as a reservoir for commensal bacteria [25]. Therefore, the pros and cons of using appendectomy for magnets retrieval should be weighed on a case-by-case basis.

## Conclusions

The ECSLA protocol is a promising tool for preventing surgical interventions and surgical complications and for possibly shortening LOS in children who have ingested multiple earth magnets.

## Data Availability

No datasets were generated or analysed during the current study.
